# Awareness of stroke, its signs, and risk factors: A cross‐sectional population‐based survey in Ghana

**DOI:** 10.1002/hsr2.2179

**Published:** 2024-06-17

**Authors:** Joseph Attakorah, Kofi Boamah Mensah, Peter Yamoah, Varsha Bangalee, Frasia Oosthuizen

**Affiliations:** ^1^ Directorate of Internal Medicine Komfo Anokye Teaching Hospital Kumasi Ghana; ^2^ Department of Pharmacy Practice Kwame Nkrumah University of Science & Technology Kumasi Ghana; ^3^ Discipline of Pharmaceutical Sciences, College of Health Science, Westville Campus University of KwaZulu‐Natal Durban South Africa; ^4^ School of Pharmacy University of Health and Allied Sciences Ho Ghana

**Keywords:** awareness, Ghana, risk factors, stroke, warning signs

## Abstract

**Background and Aims:**

The prevalence of stroke in sub‐Saharan Africa is steadily rising, leading to a growing strain on the healthcare system in the region. In the context of Ghana, stroke ranks as the third most prevalent cause of mortality. The current body of scholarly research on stroke awareness in Ghana is quite limited. The aim of this study is to assess the level of awareness of stroke, as well as its signs and symptoms among the Ghanaian population.

**Methods:**

The study employed a cross‐sectional quantitative methodology, wherein 1000 participants completed self‐administered structured questionnaires. Descriptive statistics were utilized to summarize the participants' socio‐demographic characteristics and their responses. To assess the relationship between participants' sociodemographic traits and their awareness of stroke signs, symptoms, risk factors, and perception, the Chi‐square test of independence was conducted using IBM SPSS version 26. A significance level of *p* < 0.05 was established.

**Results:**

The study identified limited awareness regarding stroke symptoms, warning signs, and risk factors. The participants exhibited an overall knowledge range of 25.9% to 47.2% concerning stroke signs and symptoms, and a range of 24%–39% regarding its causes and risk factors. Notably, paralysis (70.8%) and diet (59.9%) were the most easily recognized warning signs and risk factors for stroke, respectively. Sociodemographic characteristics such as age, religion, educational status, exposure to stroke, employment status, and marital status were shown to be associated with participants knowledge of stroke (*p* < 0.05).

**Conclusion:**

The study's results indicated a widespread lack of knowledge concerning the causes and risk factors of stroke among the Ghanaian population, highlighting the necessity for increased public education efforts to raise awareness about this condition.

## 1 INTRODUCTION

Stroke is a preventable public health issue that has become one of the leading causes of morbidity globally.^1^ Annually, 795,000 people experience a new or recurring stroke in North America.^2^ The diseṭase is the second leading cause of death worldwide and responsible for about 60% of adult‐acquired disabilities.^2^, ^3^, ^4^ Ischemic and hemorrhagic stroke represent the two distinct types of cerebrovascular diseases that impact the brain, causing a loss of brain function and, in the case of hemorrhagic stroke, the rupture of blood vessels within the brain.^5^ The disease, which was once a major burden in developed countries, has been reduced drastically due to the establishment of evidence‐based disease control measures.^2^ According to Stack et al.,^4^ the death rate from stroke in Europe has decreased over time and now accounts for 9% and 13% of deaths in men and women, respectively.^4^ Stroke has a yearly mortality rate of 5.5 million, with 75% of these deaths occurring in low to middle‐income countries.^3^, ^6^, ^7^

Neurological damages such as loss of speech, partial paralysis of certain parts of the body and numbness of the limbs are examples of signs and symptoms manifested by individuals at the onset of stroke.^3^, ^8^ Alongside hypertension, which forms the most specific risk factor for stroke, high levels of cholesterol, heart disease, smoking, diabetes, diet, and excessive use of alcohol are the well‐known risk factors for stroke.^5^, ^9^ Timely detection and treatment of stroke decrease the risk of death and disability.^2^, ^10^, ^11^

Several studies have documented a lack of knowledge of the disease, its signs, and risk factors among the general population as contributing factors to the increase in the prevalence of the disease.^12^, ^13^, ^14^ According to a study by Mosley et al., the general public's ability to recognize a stroke and take the proper action is highly correlated with understanding the condition's symptoms and risk factors.^15^ People aware of their stroke risk can encourage the intervention of stroke risk factors and lower their risk of developing a stroke.^16^

Donkor et al. suggest an increasing stroke burden in sub‐Saharan Africa.^7^ In Ghana, stroke is among the third leading causes of mortality, associated with the limited community awareness of the accompanying risk factors.^7^ Attakorah et al.^17^ report that the case fatality of stroke within the country is as high as 41%–43% in a month, while the occurrence of hypertension, the leading cause of stroke continues to rise (Sanuade et al., 2019). Studies have shown that the creation of awareness about the risk factors and symptoms of stroke can lead to a decrease in stroke incidence and help to reduce the long‐lasting impact of delayed reporting to the hospital for treatment.^18^, ^19^, ^20^ Studies about the level of awareness of stroke in Ghana are very limited. The study aims to evaluate the level of public knowledge of stroke, coupled with the associated signs and risk factors of stroke in Ghana. This will help inform the planning and structuring of public health education on stroke campaigns. Again, it will lessen the burden on the already burdened health system in the country.

## 2 METHODOLOGY

Local ethical authorization was acquired from the Kwame Nkrumah University of Science and Technology, Committee on Human Research, Publications and Ethics and the Biomedical Research Ethics Committee, South Africa, before the start of the study. A cross‐sectional study design was used to assess the knowledge, signs and symptoms, and stroke risk factors among Ghanaians at the Kejetia Central Market, Kumasi. A simple random sampling technique was used to collect data from the participants for 4 months.

## 3 STUDY SETTINGS AND SUBJECTS

The study was conducted in the Kejetia Central Market, Kumasi, in the Ashanti Region of Ghana. The Kumasi Central Market is conducted in an outdoor setting in the city of Kumasi and is the largest in West Africa. It has over 10,000 stores and stalls.^21^ Participants in this research were individuals trading or visiting the Kejetia market at the time of conducting the research.

## 4 SURVEY TOOL/INSTRUMENT

The survey tool used to collect data in this study was a well‐structured, closed‐ended questionnaire. This was developed with reference to variables and parameters reported in similar studies. The tool contained sixty‐four close‐ended questions that would be answered in about 20 min.

## 5 SAMPLE SIZE JUSTIFICATION

Given an estimated population of 1,000,000 people transact business at the study site daily and taking into consideration an assumed proportion (P) = 0.5, the table value of chi‐square (X²) for 1 degree of freedom at the desired confidence level of 95% and the degree of accuracy (d) expressed as a proportion = 0.05, the minimum sample size required to be representative of the opinions of 1,000,000 participants will be 384. The researcher, however, collected data from 1000 participants, which was used in the analysis.

## 6 DATA COLLECTION

The data was collected primarily by the principal investigator (PI) and entered electronically using Microsoft Excel 2019. The PI read the contents of the questionnaires to the respondents and obtained informed consent before administering the questionnaires. To ensure swift identification of the challenges in the data collection technique, data was solely collected by the PI so that possible changes could be made to the tool and data collection method. The questionnaire was pretested among 50 participants before the main study. The questionnaire was structured into sections to collect data on demographics, risk factors, perception and awareness of stroke. The questionnaire was then evaluated to see whether the questions were adequate to the specific objectives. After the pretesting of the questionnaire, the pilot data was analyzed for internal consistency and reliability coefficient (Cronbach's α). A Cronbach's *α* value above 0.7 was accepted and used.

## 7 DATA MANAGEMENT AND STATISTICAL ANALYSIS

To ensure data completeness and accuracy, the data collected was exported into Microsoft excel sheet and cleaned. The demographic characteristics of the participants collected were age, marital status, religion, educational level, employment history, and gender. To access participants knowledge of stroke, warning signs and risk factors, 41 questionnaire items were employed. Items to which the respondents answered utilized a three‐point response scale; 1 = Yes, 2 = No and 3 = Don't know.

## 8 RESULTS

### 8.1 Socio‐demographic characteristics of participants

A total of 1000 participants were recruited for this study. There were 414 women and 586 men. Out of these participants, majority (*n* = 300) representing 30.0% were between the ages of 20–29 years. The participants mean age was 37.62 years (95% CI: 36.7–38.5) with standard deviation of 14.5. Only 9.4% (n = 94) of the participants were above 60 years. A greater percentage of the participants were males constituting 586 (58.6%) whiles the rest were females 414 (41.4%) giving a male‐to‐female ratio of approximately 1.42:1. A total of 572 (57.2%) were Christians, 326 (32.6%) were Muslims, and traditionalists 73 (7.3%). Only 29 (2.9%) of them do not belong to any of the three major religious groups. The majority 471 (47.1%) of the participants were married followed by single or never married participants 322 (32.2%) while 27 (2.7%), 12 (1.2%) and 23 (2.3%) were divorced, separated and widowed respectively. A total of 428 (42.8%) had tertiary education followed by 306 (30.6%) with secondary education. Only 44 representing 4.4%, do not have any form of formal education.

The 633 (63.3%) of the participants were employed, while the remaining 367 (36.7%) were unemployed. Majority of the participants 505 (50.5%) do not have any family history on stroke, while the remaining 495 (41.5%) have a history of stroke in their family. A greater percentage of the participants have other associated diseases such as hypertension 506 (50.6%), diabetes 221 (22.1%), hypercholesterinemia 113 (11.3%) with only 160 of the participants representing 16% having none of the associated diseases. Majority, 597 (59.7%) of them are knowledgeable on someone with stroke while the remaining 298 (29.8%) are knowledgeable on the subject matter. Medical history of participants on whether they have or have ever had other diseases which may be associated with stroke indicates that 395 (39.5%) have had or currently have hypertension, 279 (27.9%) have had or currently have diabetes, 239 (23.9%) have had or currently have hypercholesterolemia. A greater percentage of the participants said they do not have any history of stroke, and few said they don't know if they have or have ever had stroke. Findings are presented in Table 1.

**Table 1:** Socio‐demographic characteristics of participants.

Variable	Frequency (*N* = 1000)	Percent (100.0%)
**Age range**		
<20	64	6.4
20–29	300	30
30–39	268	26.8
40–49	170	17
50–59	104	10.4
60+	94	9.4
**Gender**		
Male	586	58.6
Female	414	41.4
**Religion**		
Christianity	572	57.2
Muslim	326	32.6
Traditionalist	73	7.3
Others	29	2.9
**Marital status**		
Married	471	47.1
Single never married	322	32.2
Cohabiting	145	14.5
Divorced	27	2.7
Separated	12	1.2
Widowed	23	2.3
**Highest level of education**		
University	428	42.8
Secondary	306	30.6
Primary	222	22.2
Non formal schooling	44	4.4
**Employment history**		
Employed	633	63.3
Unemployed	367	36.7
**Family history of stroke**		
Present	495	49.5
Absent	505	50.5
**Comorbidities**		
Hypertension	506	50.6
Diabetes	221	22.1
Hypercholesterolemia	113	11.3
None of the above	160	16
**Do you know someone with stroke**		
Yes	597	59.7
No	298	29.8
I don't know	105	10.5
**Have you ever been diagnosed of stroke**		
Yes	194	19.4
No	723	72.3
I don't know	83	8.3

### 8.2 Knowledge of participants on warning signs and symptoms of stroke

Patients' responses on knowledge, warning signs, and symptoms of stroke indicated that most patients have little knowledge of stroke. When asked about the cause of stroke, 367 participants representing 36.7% said a sudden attack on the brain causes stroke, while the rest did not know the cause. Five hundred and thirty‐four (534) of the participants agreed that stroke is preventable, 185 (18.5%) said it is not preventable whiles the remaining choose ‘I don't know’. Additionally, 48.0% of respondents concurred that an individual could experience more than one stroke episode, while 34.0% expressed uncertainty about the possibility of having a stroke more than once. When asked about the effect of stroke on the daily activities of affected people, the majority of the participants, 645 (64.5%), agreed that stroke has an adverse effect on daily activities such as driving, the use of the toilet, and even the ability to have a job. The majority, 430 (43.0%), of the participants do not know the risk factors and warning signs of stroke. This was evident in their reactions to symptoms or indicators of stroke, such as dizziness, headaches, and shortness of breath, as the majority of them responded with “I'm not sure” to these signs.

The majority (69.7%) of the participants agreed that weakness on one side of the body, paralysis on one side of the body (70.8%), reduced sensation on one side of the body (63.4%), difficulty in speaking, understanding, and reading, weakness in any part of the body were severe signs of stroke. The participants, however, did not know if fainting or blackouts, collapsing, and loss of vision were signs of stroke. Findings are presented in Table 2.

**Table 2:** Participants response on knowledge, warning signs and symptoms of stroke.

Variable	Frequency (*N* = 1000)	Percent (100.0%)
**Stroke is caused by sudden attack of the brain**		
Yes	367	36.7
No	168	16.8
I don't know	465	46.5
**Stroke is preventable**		
Yes	534	53.4
No	185	18.5
I don't know	281	28.1
**Someone can have stroke more than once**		
Yes	480	48.0
No	180	18.0
I don't know	340	34.0
**Stroke has effect on daily activities like driving, use of toilet and having a job**		
Yes	645	64.5
No	176	17.6
I don't know	179	17.9
**Do you know any risk factors for stroke**		
Yes	430	43.0
No	248	24.8
I don't know	322	32.2
**Do you know any warning sign for stroke**		
Yes	470	47.0
No	232	23.2
I don't know	298	29.8
**Dizziness**		
Yes	365	36.5
No	187	18.7
I don't know	448	44.8
**Headache**		
Yes	416	41.6
No	218	21.8
I don't know	366	36.6
**Shortness of breath**		
Yes	465	46.5
No	202	20.2
I don't know	333	33.3
**Weakness on one side of the body**		
Yes	659	65.9
No	170	17.0
I don't know	171	17.1
**Paralysis on one side of the body**		
Yes	708	70.8
No	136	13.6
I don't know	156	15.6
**Numbness tingling or dead sensation of any part of the body**		
Yes	633	63.3
No	163	16.3
I don't know	204	20.4
**Numbness tingling or dead sensation one side of the body**		
Yes	634	63.4
No	185	18.5
I don't know	181	18.1
**Blurred/double/loss of vision**		
Yes	474	47.4
No	174	17.4
I don't know	352	35.2
**Sudden difficulty in speaking/understanding/reading**		
Yes	629	62.9
No	165	16.5
I don't know	206	20.6
**Weakness of any part of the body**		
Yes	697	69.7
No	149	14.9
I don't know	154	15.4
**Fainting/black out/collapse**		
Yes	451	45.1
No	223	22.3
I don't know	326	32.6

### 8.3 Overall level of knowledge of warning signs and symptoms of stroke

The participants knowledge of stroke, its warning signs and symptoms was generally inadequate. The mean score and standard deviation for the participants perception was 12.52 ± 2.1 out of a maximum score of 17 points. This led to the classification of the overall knowledge of the participants as inadequate. Scores less than fourteen (<14) were regarded as “inadequate knowledge” whiles scores from fourteen and above (≤14) were regarded as “adequate knowledge”.

Overall, only 269 (26.9%), had adequate level of knowledge, whiles 731(73.1%), had inadequate knowledge of stroke as shown in Table 3. This indicates that there is a large knowledge gap on stroke that has to be addressed by health personnel and health educators.

**Table 3:** Participants overall level on knowledge, warning signs and symptoms of stroke.

Variable	Frequency (*N* = 1000)	Percent (100.0%)
Inadequate level of knowledge	731	73.1
Adequate level of knowledge	269	26.9

### 8.4 Participants knowledge of risk factors of stroke

A greater percentage of the participants said that factors such as old age (50.7%), diabetes (50.4%), obesity (54.2%), demons or witchcraft (50.6%), excess alcohol intake (59.3%), diet (59.9%), and hypertension (54.6%) are the main causes and risk factors for stroke as stated in Table 4.

**Table 4:** Participants' response on causes and risk factors of stroke.

Variable	Frequency (*N* = 1000)	Percent (100.0%)
**Old age**		
Yes	507	50.7
No	202	20.2
I don't know	291	29.1
**Diabetes**		
Yes	504	50.4
No	206	20.6
I don't know	290	29.0
**Heart disease**		
Yes	480	48.0
No	270	27.0
I don't know	250	25.0
**Obesity**		
Yes	542	54.2
No	245	24.5
I don't know	213	21.3
**Demons**		
Yes	506	50.6
No	252	25.2
I don't know	242	24.2
**Excess alcohol intake**		
Yes	593	59.3
No	197	19.7
I don't know	210	21.0
**Diet**		
Yes	599	59.9
No	195	19.5
I don't know	206	20.6
**Hypertension**		
Yes	546	54.6
No	216	21.6
I don't know	238	23.8
**High cholesterol**		
Yes	462	46.2
No	257	25.7
I don't know	281	28.1
**Genetic (Hereditary)**		
Yes	364	36.4
No	320	32.0
I don't know	316	31.6
**Tremors**		
Yes	351	35.1
No	362	36.2
I don't know	287	28.7
**God's will**		
Yes	271	27.1
No	424	42.4
I don't know	305	30.5

### 8.5 Overall level of knowledge of causes and risk factors of stroke

The participants had inadequate knowledge of stroke causes and risk factors. The mean score and standard deviation for the participants' knowledge of stroke causes and risk factors out of a possible 12 points was 9.84 ± 2.8. This resulted in the classification of the participants' aggregate knowledge as inadequate. Below ten (10) was considered “not adequate knowledge”, while ten and above (14) was considered “adequate knowledge. Overall, 248 (24.8%) had adequate level of knowledge while 752 (75.2%) had an inadequate level of knowledge of causes and risk factors of stroke. Details are presented in Table 5.

**Table 5:** Participants overall level of knowledge of causes and risk factors of stroke.

Variable	Frequency (*N* = 1000)	Percent (100.0%)
Inadequate level of Knowledge	752	75.2
Adequate level of Knowledge	248	24.8

### 8.6 Relation between patient's demographic characteristics and their level of knowledge of stroke, its symptoms and signs

A chi‐square test of independence was performed to examine the relationship between the sociodemographic characteristics of participants and their level of knowledge of stroke, its symptoms and signs. The relationship between age, religion, marital status, highest level of education, employment history, family history of stroke, co‐morbidities, and the level of knowledge of stroke was statistically significant, χ^2^ (10, *N* = 1000) = 53.368, *p* = 0.000, χ^2^ (6, *N* = 1000) = 57.447, *p* = 0.000, χ^2^ (10, *N* = 1000) = 83.127, *p* = 0.000, χ^2^ (6, *N* = 1000) = 63.278, *p* = 0.000, χ^2^ (2, *N* = 1000) = 33.072, *p* = 0.000, χ^2^ (2, *N* = 1000) = 13.270, *p* = 0.001, χ^2^ (6, *N* = 1000) = 60.558, *p* = 0.000 respectively. These results presented in Table 6, showed that all ages, religions, marital status, level of education, employment history, family history of stroke, and co‐morbidities as presented in this study affect the level of knowledge a participant have on stroke, it's symptoms and signs. Participants' gender did not show any statistically significant association, χ^2^ (2, *N* = 1000) = 4.371, *p* = 0.112. indicating that gender of participants does not have any effect on their level of knowledge of stroke, it's symptoms and signs.

**Table 6:** Association between patient's demographic characteristics and their level of knowledge of stroke, its symptoms and signs.

Variable	Low level of knowledge	Middle level of knowledge	High level of knowledge	Total	*p* value
**Total**	**259 (25.9%)**	**472 (47.2%)**	**269 (26.9%)**	**1000 (100.0%)**	
**Age**					
<20	26 (40.6%)	27 (42.2%)	11 (17.2%)	64 (6.4%)	**0.000**
20–29	60 (20.0%)	148 (49.3%)	92 (30.7%)	300 (30.0%)	
30–39	55 (20.5%)	147 (54.9%)	66 (24.6%)	268 (26.8%)	
40–49	41 (241.1%)	78 (45.9%)	51 (30.0%)	170 (17.0%)	
50–59	32 (30.8%)	36 (34.6%)	36 (34.6%)	104 (10.4%)	
60+	45 (47.9%)	36 (38.3%)	13 (13.8%)	94 (9.4%)	
**Gender**					
Male	142 (24.2%)	273 (46.6%)	171 (29.2%)	586 (58.6%)	0.112
Female	117 (28.3%)	199 (48.1%)	98 (23.7%)	414 (41.4%)	
**Religion**					
Christianity	126 (22.0%)	271 (47.4%)	175 (30.6%)	572 (57.2%)	**0.000**
Muslim	89 (27.3%)	155 (47.5%)	82 (25.2%)	326 (32.6%)	
Traditionalist	41 (56.2%)	32 (43.8%)	0 (0.0%)	73 (7.3%)	
Others	3 (10.3%)	14 (48.3%)	12 (41.4%)	29 (2.9%)	
**Marital status**					
Married	86 (18.3%)	240 (51.0%)	145 (30.8%)	471 (47.1%)	**0.000**
Single never married	89 (27.6%)	150 (46.6%)	83 (25.8%)	322 (32.2%	
Cohabiting	76 (52.4%)	54 (37.2%)	15 (10.3%)	145 (14.5%)	
Divorced	2 (7.4%)	12 (44.4%)	13 (48.1%)	27 (2.7%)	
Separated	2 (16.7%)	6 (50.0%)	4 (33.3%)	12 (1.2%)	
Widowed	4 (17.4%)	10 (43.5%)	9 (39.1%)	23 (2.3%)	
**Highest level of education**					
University	96 (22.4%)	194 (45.3%)	138 (32.2%)	428 (42.8%)	**0.000**
Secondary	61 (19.9%)	149 (48.7%)	96 (31.4%)	306 (30.6%)	
Primary	94 (42.3%)	107 (48.2%)	21 (9.5%)	222 (22.2%)	
Non formal schooling	8 (18.2%)	22 (50.0%)	14 (31.8%)	44 (4.4%)	
**Employment history**					
Employed	129 (20.4%)	306 (58.3%)	198 (31.3%)	633 (63.3%)	**0.000**
Unemployed	130 (35.4%)	166 (45.2%)	71 (19.3%)	367 (36.7%)	
**Family history of stroke**					
Present	136 (27.5%)	206 (41.6)	153 (30.9%)	495 (49.5%)	**0.001**
Absent	123 (24.4%)	266 (52.7%)	116 (23.0%)	505 (50.5%)	
**Comorbidities**					
Hypertension	115 (22.7%)	228 (45.1%)	163 (332.2%)	506 (50.6%)	**0.000**
Diabetes	71 (32.1%)	95 (43.0%)	55 (24.9%)	221 (22.1%)	
Hyperchlolestronemia	52 (46.0%)	51 (45.1%)	10 (8.8%)	113 (11.3%)	
None of the above	21 (13.1%)	98 (61.2%)	41 (152.2%)	160 (16.0%)	
**Do you know someone with stroke**					
Yes	105 (17.6%)	282 (47.2%)	210 (35.2%)	597 (59.7%)	**0.000**
No	88 (29.5%)	152 (51.0%)	58 (19.5%)	298 (29.8%)	
I don't know	66 (62.9%)	38 (36.2%)	1 (1.0%)	105 (10.5%)	
**Have you ever been diagnosed of stroke**					
Yes	72 (371.1%)	61 (31.4%)	61 (31.4%)	194 (19.4%)	**0.000**
No	138 (19.1%)	377 (52.1%)	208 (28.8%)	723 (72.3%)	
I don't know	49 (59.0%)	34 (41.0%)	0 (0.0%)	83 (8.3%)	

### 8.7 Relation between patient's demographic characteristics and their level of knowledge of causes and risk factors of stroke

A chi‐square test of independence was performed to examine the relationship between sociodemographic characteristics of participants and their level of knowledge of causes and risk factors of stroke. The relationship between age, religion, marital status, highest level of education, employment history, previous knowledge of someone with stroke, ever diagnosed of stroke, co‐morbidities and the level of knowledge of causes and risk factors of stroke was statistically significant, χ^2^ (10, *N* = 1000) = 35.556, *p* = 0.000, χ^2^ (6, *N* = 1000) = 45.563, *p* = 0.000, χ^2^ (10, *N* = 1000) = 32.856, *p* = 0.000, χ^2^ (6, *N* = 1000) = 25.037, *p* = 0.000, χ^2^ (2, *N* = 1000) = 17.029, *p* = 0.000, χ^2^ (4, *N* = 1000) = 48.967, *p* = 0.000, χ^2^ (4, *N* = 1000) = 33.734, *p* = 0.000, χ^2^ (6, *N* = 1000) = 45.168, *p* = 0.000 respectively. These results showed that all ages, religions, marital status, highest level of education, employment history, previous knowledge of someone with stroke, ever diagnosed of stroke and co‐morbidities as presented in the study affect the level of knowledge a participant has on causes and risk factors of stroke. Participants’ gender and family history of stroke did not show any statistically significant association, χ^2^ (2, *N* = 1000) = 5.700, *p* = 0.750, χ^2^ (2, *N* = 1000) = 0.795, *p* = 0.672 respectively indicating that gender and family history of participants does not have any effect on their level of knowledge of causes and risk factors of stroke. Details are presented in Table 7.

**Table 7:** Association between patient's demographic characteristics and their level of knowledge of causes and risk factors of stroke.

Variable	Low level of knowledge	Middle level of knowledge	High level of knowledge	Total	*p* value
**Total**	**259 (25.9%)**	**472 (47.2%)**	**269 (26.9%)**	**1000 (100.0%)**	
**Age**					
<20	33 (51.6%)	24 (37.5%)	7 (10.9%)	64 (6.4%)	**0.000**
20‐29	104 (34.7%)	104 (34.7%)	92 (30.7%)	300 (30.0%)	
30‐39	88 (32.8%)	109 (40.7%)	71 (26.5%)	268 (26.8%)	
40‐49	63 (37.1%)	58 (34.1%)	49 (28.8%)	170 (17.0%)	
50‐59	33 (31.7%)	51 (49.0%)	20 (19.2%)	104 (10.4%)	
60+	45 (47.9%)	40 (42.6%)	9 (9.6%)	94 (9.4%)	
**Gender**					
Male	203 (34.6%)	223 (381%)	160 (27.3%)	586 (58.6%)	0.750
Female	163 (39.4%)	163 (39.4%)	88 (21.3%)	414 (41.4%)	
**Religion**					
Christianity	179 (31.3%)	225 (39.3%)	168 (29.4%)	572 (57.2%)	**0.000**
Muslim	131 (40.2%)	126 (38.7%)	69 (21.2%)	326 (32.6%)	
Traditionalist	47 (64.4%)	25 (34.2%)	1 (1.4%)	73 (7.3%)	
Others	9 (31.0%)	10 (34.5%)	10 (34.5%)	29 (2.9%)	
**Marital status**					
Married	150 (31.8%)	185 (39.3%)	136 (28.9%)	471 (47.1%)	**0.000**
Single never married	113 (35.1%)	126 (39.1%)	83 (25.8%)	322 (32.2%)	
Cohabiting	79 (54.5%)	51 (35.2%)	15 (10.3%)	145 (14.5%)	
Divorced	11 (40.7%)	11 (40.7%)	5 (18.5%)	27 (2.7%)	
Separated	4 (33.3%)	5 (41.7%)	3 (25.0%)	12 (1.2%)	
Widowed	9 (39.1%)	8 (34.8%)	6 (26.1%)	23 (2.3%)	
**Highest level of education**					
University	148 (34.6%)	159 (37.1%)	121 (28.3%)	428 (42.8%)	**0.000**
Secondary	104 (34.0%)	120 (39.2%)	82 (26.8%)	306 (30.6%)	
Primary	97 (43.7%)	96 (43.2%)	29 (13.1%)	222 (22.2%)	
Non formal schooling	17 (38.6%)	11 (25.0%)	16 (36.4%)	44 (4.4%)	
**Employment history**					
Employed	206 (32.5%)	247 (39.0%)	180 (28.4%)	633 (63.3%)	**0.000**
Unemployed	160 (43.6%)	139 (37.9%)	68 (18.5%)	367 (36.7%)	
**Family history of stroke**					
Present	182 (36.8%)	196 (39.6%)	117 (23.6%)	495 (49.5%)	0.672
Absent	184 (36.4%)	190 (37.6%)	131 (25.9%)	505 (50.5%)	
**Comorbidities**					
Hypertension	165 (32.6%)	204 (40.3%)	137 (27.1%)	506 (50.6%)	**0.000**
Diabetes	92 (41.6%)	84 (38.0%)	45 (20.4%)	221 (22.1%)	
Hypercholesterinemia	65 (57.5%)	39 (34.5%)	8 (8.0%)	113 (11.3%)	
None of the above	44 (27.5%)	59 (36.9%)	57 (35.6%)	160 (16.0%)	
**Do you know someone with stroke**					
Yes	181 (30.3%)	238 (39.9%)	178 (29.8%)	597 (59.7%)	**0.000**
No	125 (41.9%)	106 (35.6%)	67 (22.5%)	298 (29.8%)	
I don't know	60 (57.1%)	42 (40.0%)	3 (2.9%)	105 (10.5%)	
**Have you ever been diagnosed of stroke**					
Yes	78 (40.2%)	73 (37.6%)	43 (22.2%)	194 (19.4%)	**0.000**
No	240 (33.2%)	280 (38.7%)	203 (28.1%)	723 (72.3%)	
I don't know	48 (57.8%)	33 (39.8%)	2 (2.4%)	83 (8.3%)	

## 9 DISCUSSION

The essence of this study was to assess the awareness of stroke in the population and to evaluate their level of knowledge of the warning signs and risk factors of stroke. Overall, the study results demonstrate a low awareness of stroke signs, symptoms and risk factors. With the exception of gender, a significant association was found between socio‐demographic characteristics as presented in study and the level of knowledge of the warning signs and symptoms of stroke. The overall knowledge of the signs and symptoms of stroke was found to be low. These results are similar to a recent systematic review which reported low level of knowledge of stroke signs and symptoms among the general public in low‐middle‐income countries.^20^ Similar studies conducted in Nigeria by Rasheed & Adegbenro^8^ and Adebimpe,^22^ reported similar results of inadequate knowledge of the warning signs of stroke. This similarity in the findings may be attributed to the similar socioeconomic demographics between the two countries. However, a study conducted in Turkey, reported that most participants had a good knowledge of the signs and symptoms of stroke.^23^ A far greater level of awareness (88.2%) was recorded in Saudi Arabia as reported by Almalki et al.^24^ at King Faisal University. A possible explanation for the contrasts in findings may be due to the high level of education of participants and greater health awareness and education in those countries.

The most commonly recognized symptoms included paralysis, weakness, and a numbing sensation on one side of the body. The majority did not know they were warning signs and symptoms of stroke. The most common symptom of stroke after a study in Ethiopia^25^ was weakness in extremities, followed by sudden trouble with walking, and speech impairment, which differ from this study. Though the symptoms differ, the percentage of recognition of weakness in the extremities as a symptom of stroke is relatively consistent with this study.^25^ Contrary to the finding of this study, Hawkes et al.^10^ documented a more significant percentage (83.5%) of individuals recognizing weakness in strength as a warning sign for stroke, followed by aphasia, incoordination and headache in a study in Argentina.

The difference between the studies may be attributed to the different variables considered in the various studies and the method of analysis undertaken. Ahmed et al.,^26^ reported paralysis of one side of the body as the most recognized symptom of stroke. This is contrary to the findings of this study. In a study conducted in Jordan, it was found that the symptom of speech loss was the most widely recognized as a sign of stroke. Other symptoms like sudden weakness on one side of the body, headaches, and neck stiffness were identified by less than half of the study population.^27^

Also, in Nigeria, the authors of a study focusing on stroke awareness, warning signs, and risk factors reported sudden loss of speech as the most identified warning sign of stroke.^28^ They further reported that the participants were aware of at least one warning sign of stroke. These findings are significantly different from those of this study. Variations could be attributed to the structure of the questionnaire, the method of analysis and the experiences of people after stroke. The study found no significant association between the family history, gender, and the level of knowledge of signs and symptoms of stroke. Low awareness of the signs and symptoms of stroke in the general population will result in prehospital delays that may lead to further medical complications, which inadvertently leads to a longer and painful recovery.^20^ There is therefore a need to increase public awareness.

The study found no significant association between the family history, gender, and the level of knowledge of the cause and risk factors of stroke. The level of knowledge of the causes and risk factors were generally low. Similar to the findings of this study, Rasheed and Adegbenro^8^ also documented a low awareness of stroke risk factors in Nigeria following their research. A similar study in Indonesia also reported low level of awareness of the risk factors of stroke.^29^ The similarity may be due to shared socio‐demographic factors and health education between the two countries. Contrary to this, a higher percentage of individuals had a good level of awareness about the risk factors of stroke after the study in Turkey.^23^ A more significant proportion of individuals correctly identified more than two risk factors of stroke in a study conducted in medical school in Saudi Arabia.^24^ Workina et al.^25^ and Vincent‐Onabajo & Moses^30^ also reported a similar high awareness of stroke risk factors in their studies. These vast differences between these studies and that of this study may be attributed to the higher educational level of some of the participants. The ability to learn and research on their own, may be reasons for the difference observed. Extremely low level of awareness compared to this study was however observed in Benin after a study was carried out to assess the knowledge of stroke amongst individuals within the community (M et al., 2021). This maybe be due to the low level of health awareness and education in the country.

Again, in our study, the most identified causes and risk factors of stroke were diet, alcohol intake, hypertension, and obesity. A little over 50% of participants acknowledged old age, diabetes, and demons as causes and risk factors of stroke. A study in Iran, however, detailed hypertension and stress as the most acknowledged causes and risk factors of stroke.^31^ This was in contrast to the findings of this study. Also, Rasheed & Adegbenro^8^ also detailed that the most identified risk factor was hypertension, and the least identified was obesity after a study in Nigeria. In addition, Ahmed et al.^26^ also reported in a study done in Sudan that the most identified risk factor for stroke were hypertension, while alcohol and genetics were the least identified. Similar studies assessing the level of knowledge of the risk factors and causes of stroke, report hypertension as the most recognized risk factor (Aly, 2009).^9^, ^28^, ^30^, ^32^

Hypertension is the most identified risk factor for stroke globally.^5^ This may explain the trend of its recognition in this study as a risk factor by most participants. Contrary to the findings of this study,^7^ reported that a greater percentage (66%) of the participants could not recognize hypertension as a risk factor of stroke.^7^ These are very different from the findings of this study.

Insufficient knowledge levels recorded in this study can result in patients being unable to identify the signs, symptoms, and risk factors of stroke, and as a result, individuals could postpone obtaining medical assistance, leading to delayed treatment and worse results. Again, inadequate knowledge about the risk factors and preventive measures for stroke might contribute to a greater occurrence of risk factors such as hypertension, tobacco use, obesity, and lack of physical exercise. This can lead to a higher prevalence of strokes among the population. Insufficient awareness can lead to increased strain on healthcare systems, as a more significant number of people may need immediate medical attention for issues associated with strokes. This can exert pressure on resources and result in extended waiting periods for treatment, thereby impacting the calibre of care delivered to stroke patients.

## 10 LIMITATIONS OF STUDY

1. Since the data were self‐reported and there was no means to validate the answers, recall bias and errors on the part of the participants may lead to over‐reporting or under‐reporting.

## 11 RECOMMENDATIONS

1. Public health education programs must be designed to create more emphasis on the warning signs, symptoms and risk factors associated with stroke.
2. The Ghana Health service must liaise with the media and religious bodies to undertake public health education on stroke.
3. Lifestyle changes that can lead to reductions in the risk factors of stroke must be greatly emphasized in the media and other media platforms to help reduce the incidence of stroke in the country.

## 12 CONCLUSION

The outcome of this study shows a considerable deficit in the awareness of the signs, symptoms and risk factors of stroke in Ghana. These outcomes can lead to delayed treatment and worse results, contribute to a greater occurrence of stroke risk factors, and prevalence of strokes among the population. It is therefore essential to embark on public awareness campaigns such as multimedia campaigns using television, radio, print media, social media, and online platforms to raise awareness about stroke symptoms, risk factors, and the importance of seeking immediate medical attention. Promote policy reforms and legislative measures at the local, regional, and national levels to allocate resources for public health initiatives, enhance accessibility to stroke care services, and bolster prevention efforts. Foster partnerships with policymakers, healthcare organizations, and advocacy groups to address systemic barriers to care and prioritize stroke awareness in the country.

## AUTHOR CONTRIBUTIONS

**Joseph Attakorah**: Conceptualization; data curation; formal analysis; investigation; methodology; resources; writing—original draft; writing—review & editing. **Kofi Boamah Mensah**: Conceptualization; investigation; methodology; supervision; writing—original draft; writing—review & editing. **Peter Yamoah**: Conceptualization; formal analysis; methodology; writing—review & editing. **Varsha Bangalee**: Conceptualization; formal analysis; investigation; methodology; supervision; writing—review & editing. **Frasia Oosthuizen**: Supervision; writing—review & editing.

## CONFLICT OF INTEREST STATEMENT

The authors declare no conflict of interest. All authors have read and approved the final version of the manuscript. Joseph Attakorah had full access to all of the data in this study and takes complete responsibility for the integrity of the data and the accuracy of the data analysis.”

## TRANSPARENCY STATEMENT

The lead author Joseph Attakorah affirms that this manuscript is an honest, accurate, and transparent account of the study being reported; that no important aspects of the study have been omitted; and that any discrepancies from the study as planned (and, if relevant, registered) have been explained.

## Data Availability

Data and materials used for this study are available and will be provided upon request. If other researchers desire to request access to these data or require additional information, they should contact the author corresponding to this study.
